# Three-Dimensional Cell Culture Based on Magnetic Fields to Assemble Low-Grade Ovarian Carcinoma Cell Aggregates Containing Lymphocytes

**DOI:** 10.3390/cells9030635

**Published:** 2020-03-06

**Authors:** Caroline Natânia de Souza-Araújo, Cláudia Rodrigues Tonetti, Marcella Regina Cardoso, Liliana Aparecida Lucci de Angelo Andrade, Rodrigo Fernandes da Silva, Luís Gustavo Romani Fernandes, Fernando Guimarães

**Affiliations:** 1 School of Medicine, University of Campinas, 13083-887 Campinas, SP, Brasil; caroline.natania@yahoo.com.br (C.N.d.S.-A.); claudinha.tonetti@gmail.com (C.R.T.); macardoso86@hotmail.com (M.R.C.); lucci@unicamp.br (L.A.L.d.A.A.); rodrigoiverson@hotmail.com (R.F.d.S.); lgrf@fcm.unicamp.br (L.G.R.F.); 2 Women’s Hospital José Aristodemo Pinotti-Centro de Atenção Integral à Saúde da Mulher (CAISM), University of Campinas, 13083-881 Campinas, SP, Brasil

**Keywords:** CAISMOV24 cell line, 3D culture, cell aggregate, tumor microenvironment

## Abstract

There is a limited number of established ovarian cancer cell lines matching the low-grade serous histotype available for research purposes. Three-dimensional (3D) culture systems provide in vitro models with better tissue-like characteristics than two-dimensional (2D) systems. The goal in the study was to characterize the growth of a given low-grade serous ovarian carcinoma cell line in a 3D culture system conducted in a magnetic field. Moreover, the culture system was evaluated in respect to the assembly of malignant cell aggregates containing lymphocytes. CAISMOV24 cell line alone or mixed with human peripheral blood mononuclear cells (PBMC) were cultured using a commercially available 3D culture system designed for 24 well plates. Resulting cell aggregates revealed the intrinsic capacity of CAISMOV24 cells to assemble structures morphologically defined as papillary, and reflected molecular characteristics usually found in ovarian carcinomas. The contents of lymphocytes into co-cultured cell aggregates were significantly higher (*p* < 0.05) when NanoShuttle-conjugated PBMC were employed compared with non-conjugated PBMC. Moreover, lymphocyte subsets NK, T-CD4, T-CD8 and T-regulatory were successfully retrieved from co-cultured cell aggregates at 72h. Thus, the culture system allowed CAISMOV24 cell line to develop papillary-like cell aggregates containing lymphocytes.

## 1. Introduction

Epithelial ovarian cancer (EOC) is among the most lethal gynecological malignancies, ranking third as a cause of women’s worldwide deaths. Surgery can cure most treated women when the malignancy is still restricted to the ovaries, but 79% of women with EOC are diagnosed at advanced stages, resulting in a poor five years survival rate of 20–25% [1,2,3]. Late diagnosis is a consequence of asymptomatic initial development and an absence of specific biomarkers for the early detection of EOC. Abdominal bloating or pain are frequently the first symptoms of EOC, which are commonly associated with ascites and metastases beyond the ovaries [4,5,6]. Ninety percent of the ovarian malignancies are of epithelial origin and comprise four main histotypes. EOCs are further categorized based on their growth and molecular characteristics as type I or II. The serous histotype comprises 70% of all EOC, among which the type II or high-grade, accounts for two-thirds of all ovarian cancer deaths [7,8,9]. Although the type I or low-grade serous tumors are less frequent than high-grade tumors, women with advanced or recurrent low-grade serous carcinomas have similar survival rate as women with high-grade tumors [10,11,12]. 

Different malignant cell lines are widely employed in cancer research, and although, more than hundred established cell lines from ovarian origin are currently known, a limited number of them are well characterized as in vitro models [13,14,15,16]. Moreover, some of the cell lines commonly used as experimental models do not resemble their cognate tumor [17,18]. Finally, the majority of cell lines established from serous histotype comprises the high-grade subtype, being rare to find cell lines from low-grade subtype of serous ovarian carcinoma [10]. Thus, well-characterized cell lines, as well as culture systems that preserve the histological and molecular characteristics of ovarian neoplasms are needed, particularly, those of low-grade subtype. Our group recently established a new human EOC cell line named CAISMOV24, which spontaneously immortalized in vitro from malignant cells obtained from ascites associated with a low-grade serous adenocarcinoma of the ovary. CAISMOV24 cell line harbors the *KRAS* mutation without *TP53* mutations, which is relatively frequent in low-grade serous histotype [19]. 

Most of the studies on human EOC using in vitro models were based on two-dimensional (2D) cell cultures. Accordingly, cells of epithelial origin grow adhered to the plastic surface of the culture flask, resulting in a cell monolayer [13]. Although 2D cell culture has allowed acquisition of knowledge about tumor biology, its usefulness is limited because it does not reproduce tissue complexity, making the cells vulnerable to morphological and functional alterations. Nevertheless, three-dimensional (3D) cell cultures provide in vitro models with improved tissue-like characteristics, placing them between the in vitro 2D models and the in vivo models [20,21,22]. 3D culture furnishes in vitro models to study the interaction between malignant cells and extracellular matrix, mechanisms of malignant invasion, susceptibility and resistance of malignant cells to drugs [23,24,25,26,27]. Recently, standardized high-throughput 3D culture systems have become commercially available, raising questions on whether they would be useful for studies on the interactions of immune cells in the tumor microenvironment. 

There is a consensus that tumor infiltrating lymphocytes, as well as their cytokines, have prognostic value on ovarian cancer. Hereupon, patients with tumor infiltration of the T-CD8 subset have been associated with better treatment outcome, while the T-regulatory subset with worse responses [28,29,30,31]. All biological aspects of lymphocytes are modulated by cytokines, and cytokines such as Il-2 and IL-15 have partially overlapping properties being implicated in lymphocytes development, survival and cytotoxicity [32,33]. Although, both cytokines are primarily stimulators, the lymphocyte response to IL-2 can cause long term inhibitory effects due to activation of the T-regulatory (T-reg) lymphocyte subset, while IL-15 doesn’t have this effect [33]. Thus, our goal in this study was to characterize the growth of a given low-grade serous ovarian carcinoma cell line in a 3D culture system commercially available, which is based on the use of magnetic field to induce cell aggregation, and evaluate the culture system in respect to the assembly of malignant cell aggregates containing lymphocytes. 

## 2. Materials and Methods

### 2.1. Two and Three-Dimensional Cultures of CAISMOV24

The ovarian cancer cell line CAISMOV24 was maintained in RPMI-1640 supplemented with 10% Fetal Bovine Serum (FBS) and 2 mM L-glutamine (Nutricell, Campinas, Brazil). Two-dimensional cell cultures were carried out by seeding 10^4^ cells/cm^2^ in culture flasks (JetBiofil, Guangzhou, China). Cultures were incubated at 37 °C, 5% CO2, replenished with fresh medium every 2–3 days, and treated with trypsin/EDTA (Nutricell) for cell re-plating every 2 weeks. Three-dimensional (3D) cell cultures were carried out using the Bio-Assembler^TM^ kit designed for 24 well plates (n3D-Biosciences Inc, Houston, TX, USA). In short, NanoShuttles^TM^ were added in a T-25 flask with a ratio of 1 µL of NanoShuttles^TM^ per 20,000 cells and incubated at 37 °C and 5% CO_2_ overnight. Then, the cells were detached by treating them with 5 mL of trypsin for 5 min and washed by centrifugation (600 *g*/5 min) with balanced salt solution (PBS, Nutricell). Cell viability was determined by trypan blue (1% *w*/*v* in PBS) exclusion method and density adjusted to 10^6^ cells/mL in RPMI-1640 supplemented medium. CAISMOV24 cells conjugated with NanoShuttles^TM^ were seeded in 24-well ultralow-attachment plate (ULA, Cellstar^®^ Greiner Bio-one, Kremsmünster, Austria) at 10^5^ cells and final volume of 400 µL/well. The 3D culture was achieved by incubating (37 °C and 5% CO_2_) the plates under magnetic field, first using a bioprint drive for 3h, which was followed by a levitation drive for all culture period. This procedure promotes cells to grow as aggregates. 3D culture plate was replenished with fresh medium every 2 days until the moment of cell aggregate use. 

### 2.2. Blood Samples

The blood samples of 7 healthy donors were collected using 9mL vacuum blood-sampling tubes containing sodium heparin (Vacuette^®^, Campinas, Brazil). The peripheral blood mononuclear cells (PBMC) were isolated by gradient centrifugation, using Ficoll-Paque Plus (GE Healthcare, Uppsala, Sweden), followed by a washing procedure performed twice (centrifuged 600 *g*/5 min) using a PBS. Cell numbers were assessed in a Neubauer chamber using acetic acid solution (2% in PBS) and the trypan blue exclusion method to assess viability. 

### 2.3. Three-Dimensional Co-Cultures of CAISMOV24 and Lymphocytes

PBMCs were conjugated with NanoShuttles^TM^ by mixing them at a proportion of 20,000 viable cells to 1 µL of the nanoparticle in a conical tube. Subsequently, PBMCs suspension were subjected to three cycles of centrifugation and resuspension (30 *g*/5 min), by pipetting the cells up and down (50 times), without changing the medium. CAISMOV24 cells were conjugated to NanoShuttle^TM^ as aforementioned. NanoShuttle^TM^ conjugated PBMCs and CAISMOV24 cells were seeded at 1:5 cell ratio in 24-well ultralow-attachment plate at 1.2 × 10^5^ cells and final volume of 400 µL/well. 3D culture was achieved by incubating (37 °C and 5% CO_2_) the plates under magnetic field, first using a bioprint drive for 3h, which was followed by a levitation drive for all culture period. Co-cultured cell aggregates were either treated with IL-15 (60 ng/mL final concentration/well) daily or not. Additionally, culture plates also had wells containing PBMCs conjugated with NanoShuttel^TM^ alone, either treated with IL-15 or not, as experiment controls. Additionally, 3D co-cultures were carried out using PBMCs labelled with carboxyfluorescein-succinimydil-ester (CFSE, Molecular Probes, Invitrogen, Burlington, ON, Canada). For cell labelling, PBMCs were incubated in 3 µM CFSE-PBS solution at a density of 2.5 × 10^5^ cells/mL for 15 min at 37 °C. 

### 2.4. Histological Analysis and Immunohistochemisry

Cell aggregates of CAISMOV24 were either submitted to cryo-sections (Cryostat CM 1850, Leica Biosystems, Wetzlar, Germany) intended for immunohistochemistry analysis or fixed in 4% formalin and routinely processed to obtain histological sections from paraffin-embedded tissue. Briefly, immunohistochemistry was carried out from a series of consecutive cryosections (4 μm) placed on silanized slides, which were fixed with acetone for 15 min, washed in PBS and incubated in appropriate dilutions of primary antibodies (PAX8 clone BC12, control no. 901-438-070919, dil. 1:100; estrogen receptor clone 1D5, control no. 901-054-081817, dil. 1:200; Progesterone receptor clone 16, control no. 903-424-020818, dil. 1:200, Biocare Medical, Pacheco, CA, USA) and secondary peroxidase conjugated-antibody, following standard procedures of the Anatomopathology Department at the University of Campinas Hospital. Additionally, cell aggregates resulting from the co-culture of CAISMOV24 cells with CFSE-labelled PBMCs were submitted to cryo-sections. Cell aggregate cuts on slides were fixed with acetone for 15 min, washed with PBS, incubated in permeabilization solution (0.0387 M Na_3_C_6_O_7_ with 1% Triton X-100) for 2 min, washed again, and finally, stained with 4’,6-Diamidine-2’-phenylindole dihydrochloride/5 min (DAPI, Roche Diagnostics GmbH, Mannheim, Germany). Cuts on slide were covered with mounting medium and microscope slip. Microscope examination was carried out in fluorescence microscopy (Eclipse 80i, Nikon^®^, Tokyo, Japan; with LP 430 nm and 510 nm filters). 

### 2.5. Lymphocyte Phenotyping

Lymphocytes were recovered from the co-cultured cell aggregates and phenotyped for the identification of their subsets. For this procedure, six cell aggregates obtained under the same culture conditions were grouped to be processed together for cell disaggregation by up and down pipetting in staining solution (PBS with 2% SFB and 0.2 mM EDTA). The resulting single cell suspension was washed by centrifugation, and the final cell pellet suspended with staining solution to adjust cells density to 10^6^ cells/mL. A flow cytometric-based assay was conducted according to standard procedures. Briefly, the 0.3 × 10^5^ cells were mixed with 50 μL of staining solution containing a mix of fluorophore-conjugated monoclonal antibodies at a 1:50 dilution: anti-CD3 APC-H7 (clone SK7), anti-CD4 PerCP-Cy5.5 (clone SK3), anti-CD25 BB515 (clone 2A3), anti-CD56 PE-Cy7 (clone B159), anti-CD127 Alexa Fluor647 (clone HIL-7R-M21) and anti-CD8 BV421 (clone 3G8) (BD Pharmingen™, San Jose, CA, USA). Cells were incubated for 30 min on ice and protected from light. After the incubation, cells were washed twice with PBS and the final pellets suspended with 300 µL PBS for acquisition on a FACS Verse flow cytometer using the FACSuite software (Becton Dickinson, San Jose, CA, USA). The FlowJo software was used for data analysis. The lymphocyte population was identified by the FSC and SSC parameters, and the FSC-Area vs. FSC-Height was used to eliminate doublets. Within the lymphocyte population, the CD3^+^ lymphocytes were identified by anti-CD3 APC-H7. Within the CD3^+^ lymphocytes, CD4^+^ and CD8^+^ populations were identified as well. Within the CD4^+^ population, the T-reg population was identified by plotting anti-CD25 BB515 vs. anti-CD127 Alexa Fluor647.

### 2.6. CAISMOV24 2D and 3D in Vitro Growth Kinetics

Cell division of CAISMOV24 cells in 2D and 3D cultures was assessed by flow cytometry. For this end, CAISMOV24 cells were labelled with violet proliferation dye 450 (VPD450, BD Horizon™, San Diego, CA, USA) according to the manufacturer’s instructions, prior being cultivated under 2D or 3D culture systems. At day five of 3D culture four to six cell aggregates of CAISMOV24 were grouped to be processed together for cell disaggregation. Simultaneously, CAISMOV24 cells from 2D culture flasks were treated with trypsin/EDTA for detachment and cell disaggregation. Subsequently, 2D and 3D cell suspensions were acquired in a FACS Verse with FACS Suite software. Cell suspensions were analyzed by setting the appropriate SSC-A/FSC-A gate on tumor cells and considering the fluorescence intensities on day 0 in VPD450 labelled and unlabeled cells. The proliferation platform of FlowJo software was used for the data analysis. The proliferation index was calculated by dividing the total number of divisions by the number of cells that underwent at least one division. 

### 2.7. Statistics and Calculations

Multi-comparison analysis of variables was performed by ANOVA followed by a post hoc multiple comparison test. The level of significance was set at *p*-value < 0.05. The ratio of lymphocytes retrieved from cell aggregates was calculated by dividing the number of events detected within the SSC-A/FSC-A gate of CAISMOV24 cells by the number of events within the SSC-A/FSC-A gate of lymphocytes. 

## 3. Results

### 3.1. 3D cultures and Proliferation Assays

**Figure 1:**
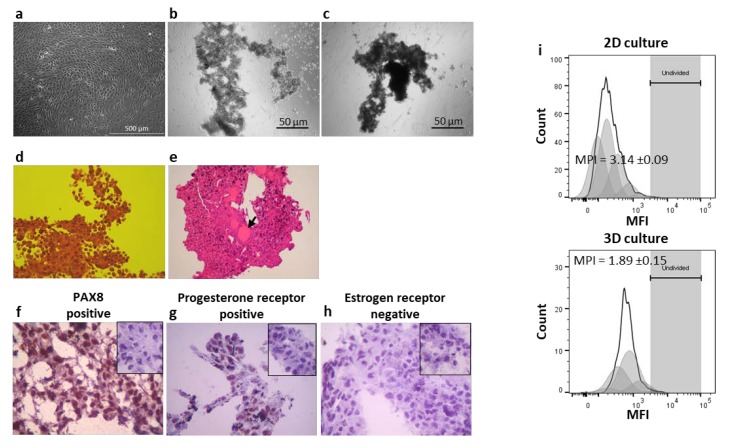
Analysis of morphological and histological features of the CAISMOV24 cells in 3D culture. Phase contrast microscopy of CAISMOV24 cells growing as (**a**) monolayer in 2D culture or growing as cell aggregates in 3D culture at (**b**) 24 h and (**c**) at 72 h. (**d** and **e**) Brightfield microscopy of histological cuts representative of CAISMOV24 cell aggregates (*n* = 8); (**d**) Histological cut showing papillary morphology of the cell aggregate (obj. 40×; H&E staining) and (**e**) the presence of focal acinar arrangement with secreted material (arrow; obj. 10×; H&E staining). Immunohistochemistry analysis of cryosections of 3D-cultured CAISMOV24 cells (*n* = 6 cell aggregates) showing nuclear expression of PAX8 (**f**) and progesterone receptor (**g**) in brown, as well as, absence of estrogen receptor (**h**) compared with their respective negative controls (insets); cells are counterstained with hematoxylin. (**i**) Proliferation profile of CAISMOV24 cells assessed by flow cytometry on day 5, following cell labeling with violet proliferation dye 450 (VPD450); shaded areas represent each of the new cell generations, which retained approximately half of the VPD450 fluorescence intensity of their parent cells. The mean proliferation index of CAISMOV24 cells was significant lower (*p* < 0.0001, t-student test) in 3D culture (*n* = 7 experimental repetitions) than in the 2D culture (*n* = 3). MPI = mean proliferation index.

Three dimensional cultures of CAISMOV24 cell line was followed by phase contrast microscopy and representative images are depicted in Figure 1. The first 3 h under magnetic field promoted by the bioprint drive brought CAISMOV24 cells together in a round-shaped structure. Subsequently, under magnetic field of the levitation drive, the initial rounded structure evolved irregularly, generating regions with variable amounts of aggregated cells and spindle-like elongated structures (Figure 1b, 24 h). The final arrangement of CAISMOV24 cells was morphologically defined as papillary, as revealed by histological analysis of the cell aggregates (Figure 1d). Histological cuts also revealed the presence of focal acinar arrangement with secreted material, pointing out that 3D culture system enabled CAISMO24 cells to evolve glandular-like structures (Figure 1e). Finally, histological cuts of CAISMOV24 cell aggregates assessed by immunohistochemistry revealed diffuse nuclear expression of PAX8 and progesterone receptor, as well as absence of estrogen receptor expression (Figure 1f–h respectively). VPD450-stained CAISMOV24 cells assessed by flow cytometry showed that mean proliferation index of the cells maintained in 3D cultures (1.87 ± 0.15 times, *n* = 7) was significantly lower (*p* < 0.0001) than in the 2D cultures (3.14 ± 0.09 times, *n* = 3) (Figure 1i). 

### 3.2. CAISMOV24 Cell Aggregate Contents of Lymphocytes

Three dimensional co-cultures of CAISMOV24 cells with PBMCs (5:1 cell ratio respectively) resulted in aggregates of malignant cells containing lymphocytes. These cell aggregates evolved similarly to what was previously described for aggregates of CAISMOV24 cells alone. Moreover, PBMCs conjugated with NanoShuttle, which were maintained alone under the same 3D culture conditions for comparison purposes, showed the inability of leukocytes to generate aggregates. 

**Figure 2:**
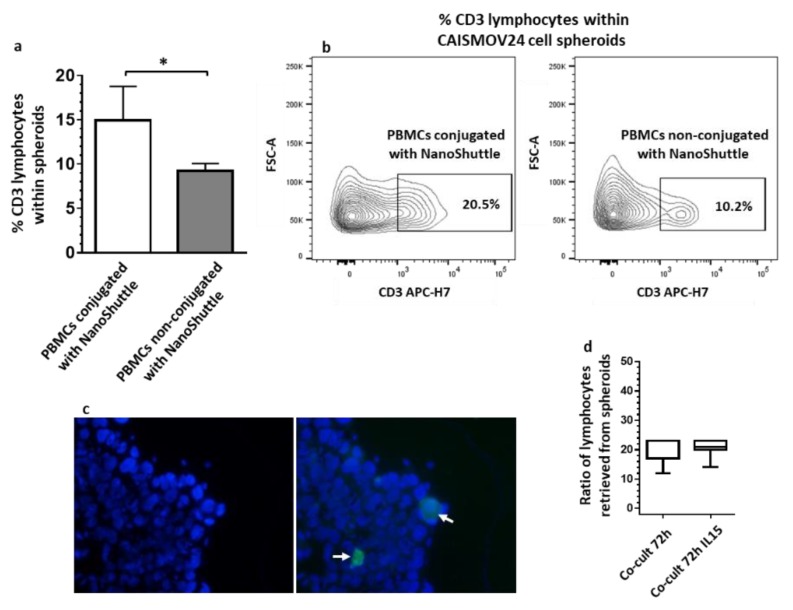
Analysis of the contents of lymphocytes in CAISMOV24 cell aggregates obtained by 3D co-culture of CAISMOV24 cells with PBMCs. (**a**) 3D co-cultures performed with NanoShuttle-conjugated PBMCs (*n* = 6 experimental repetitions) have significant (* *p* < 0,05) higher percentages of CD3 lymphocyte subset than co-cultures performed with PBMCs non-conjugated with NanoShuttle (*n* = 3 experimental repetitions). (**b**) Contour plots from a representative assay performed with NanoShuttle-conjugated and non-conjugated PBMCs from the same blood sample. (**c**) Fluorescence microscopy from a representative cryo-section of cell aggregates showing CFSE-labelled PBMCs (arrow). (**d**) The ratio of lymphocytes retrieved from cell aggregates is similar between co-cultures at 72 h that were supplemented or not with IL-15 (*n* = 7 experimental repetitions).

The contents of lymphocytes within CAISMOV24 cell aggregates were assessed both by cell disaggregation followed by flow cytometry analysis as well as fluorescence microscopy. It was observed that co-cultures performed with NanoShuttle-conjugated PBMCs had significantly higher (*p* < 0.05) percentages of CD3+ lymphocytes than those with non-conjugated PBMCs (Figure 2a,b). The presence of leukocytes within CAISMOV24 cell aggregates were confirmed by fluorescence microscopy of cryo-sections obtained from cell aggregates containing CFSE-labelled PBMCs (Figure 2c). The ratio of lymphocytes retrieved from cell aggregates were approximately 20:1 (CAISMOV24: lymphocyte) 72 h after co-culture initiation, being similar between in vitro cultures that were supplemented or not with IL-15 (Figure 2d).

**Figure 3:**
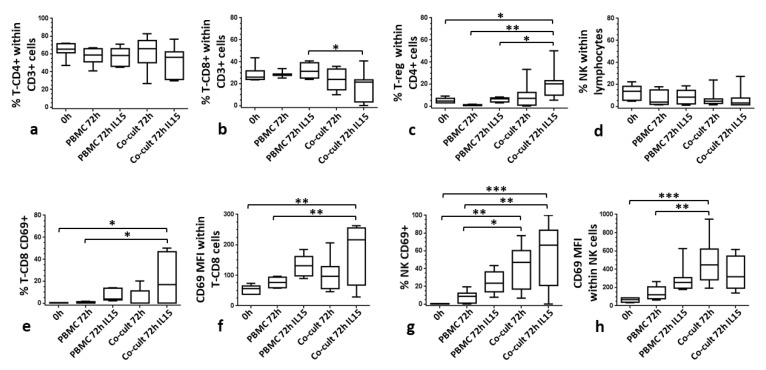
Comparison of the contents of lymphocyte subsets in CAISMOV24 cell aggregates obtained by 3D co-culture of CAISMOV24 cells with PBMCs. (**a**), (**b**) and (**c**) frequencies of T lymphocytes subsets T-CD4, T-CD8 and T-reg respectively, as well as (**d**) NK lymphocytes. (**b**) T-CD8+ subset is significant lower (*p* < 0.05) in CAISMOV24 cell aggregates supplemented with IL-15 compared with PBMCs maintained in the same culture conditions. (**c**) T-reg subset is significantly higher (*p* < 0.05) in CAISMOV24 cell aggregates supplemented with IL-15 compared to PBMCs cultures. CD69 molecule is upregulated on NK and CD8 lymphocytes retrieved from CAISMOV24 cell aggregates. (**e**,**f**) CD69 upregulation on CD8+ lymphocytes is associated with supplementation of the culture with IL-15, while (**g**,**h**) on NK lymphocytes it was associated with presence of CAISMOV24 cells. Values were presented as whisker plots and medians (*n* = 7 experimental repetitions with different blood donors); statistical analyses were performed by ANOVA followed by Tukey’s multiple comparisons test. Significant statistical differences are indicated with * (* *p* < 0.05, **p < 0.01 and *** *p* < 0.001).

Furthermore, lymphocyte subsets were further assessed. Thus, Figure 3 shows the proportions of NK (CD3^−^ CD56^+^) lymphocytes, as well as T lymphocytes and their subsets, T-CD4^+^ (CD3^+^CD4^+^), T-CD8^+^ (CD3^+^CD8^+^) and T-reg (CD4^+^CD25^+^CD127^−^). Although, no significant differences were observed in the percentages of T-CD4^+^ and NK lymphocytes among the different culture conditions (Figure 3a,d), proportions of the T-CD8^+^ subset within the CD3^+^ lymphocytes decreased in CAISMOV24 cell aggregates, being significantly (*p* < 0.05) lower in co-cultures supplemented with IL-15 compared with PBMCs maintained in the same culture conditions (Figure 3b). In addition, the proportion of the T-reg subset within the CD4^+^ population was significantly higher (*p* < 0.05) in CAISMOV24 cell aggregates supplemented with IL-15 compared to PBMCs cultures (Figure 3c). Finally, it was observed an upregulation of the CD69 molecule on NK and CD8 lymphocytes retrieved from CAISMOV24 cell aggregates. In this context, CD69 upregulation on CD8^+^ lymphocytes was associated with supplementation of the culture with IL-15 (Figure 3e,f), while on NK lymphocytes it was associated with presence of CAISMOV24 cells (Figure 3g,h). 

## 4. Discussion

Studies using 3D cell cultures have increased over the past years. This fact is not only a consequence of the improved accuracy delivered by this cell culture approach, but it is also a result of the availability of new, simplified and high-throughput protocols. In this context, the present study successfully evaluated a commercially available 3D culture system to assemble low-grade serous ovarian carcinoma cell aggregates containing lymphocytes. 

Frequently, 3D culture models of neoplasms, including EOC, are achieved by culturing tissue fragments, suspension of primary cells or cell lines upon a natural or synthetic polymer matrix [23,24,25,26,27,34,35]. Another way to generate 3D cultures of malignant cells is based on the use of ULA containers that prevent cell adhesion, which combined with suitable culture media and/or agitation promote the growth of epithelial cells as free cell aggregates [36,37,38]. New 3D cell culture technologies have combined magnetic field with ULA containers to promote aggregation of the cells that were previously conjugated with nanoparticles containing iron. This is the case of the n3D-Bioscieces culture system, which was successfully employed to generate cell aggregates of different cell types, including stem and primary cells from humans, as well as other human malignant cells [39,40,41]. 

In regard to ovarian cancer cell lines, Lee and coworkers [42] used 31 cell lines to compare a 3D culture system based on synthetic polymer matrix with the 2D culture. Heredia-Soto and coworkers [43] recently standardized a 3D culture system employing ULA containers to generate cell aggregates with 16 cell lines. These studies included OAW42 and PEO16 respectively as the only cell lines of low-grade serous histotype. Finally, Pan and coworkers [44] employed the n3D-Bioscieces system based on magnetic field to evaluate the influence of microRNA on cell aggregation of six different ovarian cancer cell lines, all of them of high-grade. Cell aggregates obtained in these studies varied in their morphology, from round to irregular-elongated, and cell compaction, from dense to loose, showing that the 3D culture systems did not determine alone the final shape of the cell aggregates. Thus, as stated by Lee and coworkers, 3D culture allows cell lines to reveal certain histological differentiation, even after prolonged culture in 2D [42]. Correspondingly, our results suggested that aggregates of CAISMOV24 cells were shaped not only by the magnetic field, but also by the intrinsic capacity of the growing cells to organize their final arrangement. As a result, cell aggregates observed in our 3D cultures were consistent with the cytological pattern found in peritoneal lavages or ascites from patients with ovarian cancer, which are positive for the presence of malignant cells [45]. Moreover, histological analysis showed that CAISMOV24 cell aggregates displayed papillary morphology, and molecular phenotype consistent with functional EOC cells, particularly, it was detected nuclear expression of PAX8 molecule, which is a biomarker frequently reported occurring in low-grade serous ovarian cancer [35]. 

Similar to what was previously reported for other EOC cell lines [42,46], we observed that CAISMOV24 cell had a lower cell proliferation index in 3D culture compared with 2D. Such a decrease in cell proliferation would most likely be a consequence of inhibition by cell contact, which happens earlier in 3D cultures than in 2D. However, whether magnetic field would play a role on cell proliferation inhibition remain to be assessed. Although, studies have stated that weak magnetic field do not produce biological effects [39,47,48], there are data suggesting that exposure to magnetic field could specifically target highly proliferative cell populations, such as malignant cells [49,50].

Differently from 3D culture employing ULA plates alone, we hypothesized that since the Bio-Assembler^TM^ kit combines magnetic field with ULA plates, it could enable to assembly of EOC cell aggregates containing lymphocytes. Correspondingly, our results showed that co-culture of CAISMOV24 cells with NanoShuttle^TM^-conjugated PBMCs under magnetic field boosts significantly the contents of lymphocytes within EOC cell aggregates. Moreover, we demonstrated the feasibility of accessing different lymphocyte subsets within EOC cell aggregates, validating this 3D culture system as a useful in vitro approach to address lymphocyte interactions in EOC microenvironment. 

Our results showed that cytotoxic cells (NKs and CD8 lymphocytes) were activated, and pointed the T-reg subtype as a long-term persistent lymphocyte present into the cell aggregates, in a sense similar to what have been shown in patients with EOC [51,52].

## 5. Conclusions

We conclude that the 3D culture system allowed CAISMOV24 cell line to develop papillary-like cell aggregates. The culture system also allowed retrieval of lymphocytes from cell aggregates obtained by co-culture of PBMCs and CAISMOV24 cell line. Thus, we assumed this 3D culture system suitable for the study of immune cell interactions in tumor microenvironment.

## References

[1] Ferlay J., Soerjomataram I., Dikshit R., Eser S., Mathers C., Rebelo M., Parkin N.M., Forman D., Bray F. (2014). Cancer incidence and mortality worldwide: Sources, methods and major patterns in GLOBOCAN 2012. Int. J. Cancer.

[2] Vargas A.N. (2014). ecancermedicalscience. Ecancermedicalscience.

[3] Siegel R.L., Miller K.D., Jemal A. (2017). Cancer statistics, 2017. CA Cancer J. Clin..

[4] Naora H., Montell D.J. (2005). Ovarian Cancer Metastasis: Integrating insights from disparate model organisms. Nat. Rev. Cancer.

[5] Huang H., Li Y., Lan C.Y., Huang Q.D., Feng Y.L., Huang Y.W., Liu J.H. (2013). Clinical significance of ascites in epithelial ovarian cancer. Neoplasma.

[6] Kim S., Kim B., Song Y.S. (2016). Ascites modulates cancer cell behavior, contributing to tumor heterogeneity in ovarian cancer. Cancer Sci..

[7] Ferreira P.A.R., Sallum L.F.T.A., Sarian L.O., Andrade L.A.L.D.A., Derchain S. (2012). Carcinoma de ovário seroso e não seroso: Tipo histológico em relação ao grau de diferenciação e prognóstico. Rev. Bras. Ginecol. Obstet. RBGO Gynecol. Obstet..

[8] Berns E.M.J.J., Bowtell D. (2012). The Changing View of High-Grade Serous Ovarian Cancer. Cancer Res..

[9] Kurman R.J.,  Shih I. (2016). The Dualistic model of ovarian carcinogenesis revisited, revised, and expanded. Am. J. Pathol..

[10] Fernández M.L., DiMattia G.E., Dawson A., Bamford S., Anderson S., Hennessy B.T., Anglesio M.S., Shepherd T.G., Salamanca C., Hoenisch J. (2016). Differences in MEK inhibitor efficacy in molecularly characterized low-grade serous ovarian cancer cell lines. Am. J. Cancer.

[11] Okoye E., Euscher E.D., Malpica A. (2016). Ovarian Low-grade Serous Carcinoma. Am. J. Surg. Pathol..

[12] Ahn G., Folkins A.K., McKenney J.K., Longacre T.A. (2016). Low-grade Serous Carcinoma of the Ovary. Am. J. Surg. Pathol..

[13] Thériault B.L., Portelance L., Mes-Masson A.-M., Nachtigal M. (2013). Establishment of Primary Cultures from Ovarian Tumor Tissue and Ascites Fluid. Adv. Struct. Saf. Stud..

[14] Chen J., Wang J., Zhang Y., Chen D., Yang C., Kai C., Wang X., Shi F., Dou J. (2014). Observation of ovarian cancer stem cell behavior and investigation of potential mechanisms of drug resistance in three-dimensional cell culture. J. Biosci. Bioeng..

[15] Giri S., Rattan R., Deshpande M., Maguire J.L., Johnson Z., Graham R.P., Shridhar V. (2014). Preclinical Therapeutic Potential of a Nitrosylating Agent in the Treatment of Ovarian Cancer. PLoS ONE.

[16] Jacob F., Nixdorf S., Hacker N.F., Heinzelmann-Schwarz V. (2014). Reliable in vitro studies require appropriate ovarian cancer cell lines. J. Ovarian Res..

[17] Barretina J., Caponigro G., Stransky N., Venkatesan K., Margolin A.A., Kim S., Wilson C., Lehár J., Kryukov G., Sonkin D. (2018). Addendum: The Cancer Cell Line Encyclopedia enables predictive modelling of anticancer drug sensitivity. Nature.

[18] Domcke S., Sinha R., Levine D.A., Sander C., Schultz N. (2013). Evaluating cell lines as tumour models by comparison of genomic profiles. Nat. Commun..

[19] Da Silva R., Cardozo D.M., Rodrigues G.O.L., De Souza-Araújo C.N., Migita N.A., Andrade L.A.L.D.A., Derchain S., Yunes J.A., Guimaraes F. (2017). CAISMOV24, a new human low-grade serous ovarian carcinoma cell line. BMC Cancer.

[20] Kim J. (2005). Three-dimensional tissue culture models in cancer biology. Semin. Cancer Boil..

[21] Yamada K.M., Cukierman E. (2007). Modeling Tissue Morphogenesis and Cancer in 3D. Cell.

[22] Freire D. (2017). Os jardins suspensos das células. Pesqui. FAPESP.

[23] Xu F., Celli J., Rizvi I., Moon S., Hasan T., Demirci U. (2011). A three-dimensional in vitro ovarian cancer coculture model using a high-throughput cell patterning platform. Biotechnol. J..

[24] Loessner D., Holzapfel B.M., Clements J.A. (2014). Engineered microenvironments provide new insights into ovarian and prostate cancer progression and drug responses. Adv. Drug Deliv. Rev..

[25] Augustine T., Dix-Peek T., Duarte R., Candy G.P. (2015). Establishment of a heterotypic 3D culture system to evaluate the interaction of TREG lymphocytes and NK cells with breast cancer. J. Immunol. Methods.

[26] Hirt C., Papadimitropoulos A., Mele V., Muraro M.G., Mengus C., Iezzi G., Terracciano L., Martin I., Spagnoli G.C. (2014). “In vitro” 3D models of tumor-immune system interaction. Adv. Drug Deliv. Rev..

[27] Aihara A., Abe N., Saruhashi K., Kanaki T., Nishino T. (2016). Novel 3-D cell culture system forin vitroevaluation of anticancer drugs under anchorage-independent conditions. Cancer Sci..

[28] Gavalas N.G., Karadimou A., Dimopoulos M.A., Bamias A. (2011). Immune Response in Ovarian Cancer: How Is the Immune System Involved in Prognosis and Therapy: Potential for Treatment Utilization. Clin. Dev. Immunol..

[29] Hwang W.-T., Adams S.F., Tahirovic E., Hagemann I., Coukos G. (2011). Prognostic significance of tumor-infiltrating T cells in ovarian cancer: A meta-analysis. Gynecol. Oncol..

[30] Curiel T.J., Coukos G., Zou L., Alvarez X., Cheng P., Mottram P., Evdemon-Hogan M., Conejo-Garcia J.R., Zhang L., Burow M. (2004). Specific recruitment of regulatory T cells in ovarian carcinoma fosters immune privilege and predicts reduced survival. Nat. Med..

[31] Ghiringhelli F., Meénard C., Terme M., Flament C., Taieb J., Chaput N., Puig P.E., Novault S., Escudier B., Vivier E. (2005). CD4+CD25+ regulatory T cells inhibit natural killer cell functions in a transforming growth factor–β–dependent manner. J. Exp. Med..

[32] Huntington N.D. (2014). The unconventional expression of IL-15 and its role in NK cell homeostasis. Immunol. Cell Boil..

[33] Fehniger T.A., Cooper M., Caligiuri M.A. (2002). Interleukin-2 and interleukin-15: Immunotherapy for cancer. Cytokine Growth Factor Rev..

[34] White E.A., Kenny H.A., Lengyel E. (2014). Three-dimensional modeling of ovarian cancer. Adv. Drug Deliv. Rev..

[35] Kopper O., De Witte C.J., Lõhmussaar K., Valle-Inclan J.E., Hami N., Kester L., Balgobind A.V., Korving J., Proost N., Begthel H. (2019). An organoid platform for ovarian cancer captures intra- and interpatient heterogeneity. Nat. Med..

[36] Yuhas J.M., Li A.P., Martinez A.O., Ladman A.J. (1997). A simplified method for production and growth of multicellular tumor spheroids. Cancer Res..

[37] Casey R.C., Burleson K.M., Skubitz K.M., Pambuccian S.E., Oegema T.R., Ruff L.E., Skubitz A.P.N. (2001). β1-Integrins Regulate the Formation and Adhesion of Ovarian Carcinoma Multicellular Spheroids. Am. J. Pathol..

[38] Weiswald L.-B., Bellet D., Dangles-Marie V. (2015). Spherical cancer models in tumor biology. Neoplasia.

[39] Haisler W.L., Timm D.M., Gage J.A., Tseng H., Killian T.C., Souza G.R. (2013). Three-dimensional cell culturing by magnetic levitation. Nat. Protoc..

[40] Guo W.M., Loh X.J., Tan E.Y., Loo J., Ho V.H.B. (2014). Development of a Magnetic 3D Spheroid Platform with Potential Application for High-Throughput Drug Screening. Mol. Pharm..

[41] Du V., Luciani N., Richard S., Mary G., Gay C., Mazuel F., Reffay M., Ménasché P., Agbulut O., Wilhelm C. (2017). A 3D magnetic tissue stretcher for remote mechanical control of embryonic stem cell differentiation. Nat. Commun..

[42] Lee J.M., Mhawech-Fauceglia P., Lee N., Parsanian L.C., Lin Y.G., Gayther S.A., Lawrenson K. (2013). A three-dimensional microenvironment alters protein expression and chemosensitivity of epithelial ovarian cancer cells in vitro. Lab. Investig..

[43] Heredia-Soto V., Redondo A., Berjón A., Miguel-Martín M., Díaz E., Crespo R., Hernández A., Yébenes L., Gallego A., Feliu J. (2018). High-throughput 3-dimensional culture of epithelial ovarian cancer cells as preclinical model of disease. Oncotarget.

[44] Pan Y., Robertson G., Pedersen L., Lim E., Hernandez-Herrera A., Rowat A.M., Patil S.L., Chan C.K., Wen Y., Zhang X. (2016). miR-509-3p is clinically significant and strongly attenuates cellular migration and multi-cellular spheroids in ovarian cancer. Oncotarget.

[45] Shield P. (2014). Peritoneal washing cytology. Cytopathology.

[46] Lawrenson K., Notaridou M., Lee N., Benjamin E., Jacobs I., Jones C., Gayther S.A. (2013). In vitro three-dimensional modeling of fallopian tube secretory epithelial cells. BMC Cell Boil..

[47] Tseng H., Gage J.A., Raphael R.M., Moore R.H., Killian T.C., Grande-Allen K.J., Souza G.R. (2013). Assembly of a Three-Dimensional Multitype Bronchiole Coculture Model Using Magnetic Levitation. Tissue Eng. Part C Methods.

[48] Hore P.J. (2012). Are biochemical reactions affected by weak magnetic fields?. PNAS.

[49] Van Huizen A.V., Morton J.M., Kinsey L.J., Von Kannon D.G., Saad M.A., Birkholz T.R., Czajka J.M., Cyrus J., Barnes F.S., Beane W.S. (2019). Weak magnetic fields alter stem cell–mediated growth. Sci. Adv..

[50] Martino C.F., Portelli L., McCabe K., Hernandez M., Barnes F. (2010). Reduction of the Earth’s magnetic field inhibits growth rates of model cancer cell lines. Bioelectromagnetics.

[51] Landskron J., Helland Ø, Torgersen K.M., Aandahl E.M., Gjertsen B.T., Bjørge L., Tasken K. (2014). Activated regulatory and memory T-cells accumulate in malignant ascites from ovarian carcinoma patients. Cancer Immunol. Immunother..

[52] Da Silva R., Yoshida A., Cardozo D.M., Jales R.M., Paust S., Derchain S., Guimaraes F. (2017). Natural Killer Cells Response to IL-2 Stimulation Is Distinct between Ascites with the Presence or Absence of Malignant Cells in Ovarian Cancer Patients. Int. J. Mol. Sci..

